# How Does the Interactivity of Social Media Affect the Adoption of New Green Products?

**DOI:** 10.3389/fpsyg.2021.786372

**Published:** 2021-11-25

**Authors:** Xin Cao, Xitong Wu, Xiaozhi Huang

**Affiliations:** ^1^School of Economics, Guangxi University, Nanning, China; ^2^School of Economics and Trade, Guangxi University of Finance and Economics, Nanning, China; ^3^School of Business Administration, Guangxi University, Nanning, China

**Keywords:** green newsfeed advertising, sociality, receptivity to green advertising, green involvement, adoption of new green products

## Abstract

In the era of mobile internet, newsfeed advertising, which is most applicable to consumption scenarios that involve mobile devices, has become a core driving force in advertising. The rapid advancement of technology with respect to newsfeed advertising has not only reshaped the green consumption sector but has also had significant impacts on consumer psychology and behavior. When faced with highly social green newsfeed advertising, consumers are receptive to different degrees, which ultimately affects their receptivity to new green products. Through an experiment and a questionnaire, we find the following: (1) Consumers prefer new green products advertised through high-sociality green newsfeed advertisements more than those advertised with low-sociality ads. (2) Receptivity to green advertising can mediate the impact of the sociality of newsfeed advertising on consumer adoption of new green products. (3) The aforementioned direct effect and mediating effect are also affected by consumers’ green involvement. Our paper has both theoretical and practical significance; that is, we contribute to the research on the impacts of the sociality of green advertising on the psychological mechanisms underlying consumer adoption of new green products. Additionally, we provide managerial recommendations for the future development of green newsfeed advertising and for improvements in consumer impressions of green consumption.

## Introduction

In the era of mobile internet, the traditional vertical communication paradigm, which is dominated by enterprises, is fading. More enterprises have begun to adopt a horizontal communication paradigm, which is customer-centric and more applicable to consumption scenarios that involve mobile devices (Wollschlaeger et al., 2017). Newsfeed advertising has emerged during this historic moment, developed rapidly, and become the new driving force behind product promotion for many enterprises (Grewal et al., 2016). Newsfeed advertising is advertising embedded within the information that streams over media platforms. Moreover, newsfeed advertising can be mutually integrated into the platforms (nativity), be presented line by line according to the presentation mode of the platform (dynamism), and support consumer participation (sociality; Huang Minxue, 2019). With the development of Web 2.0, the interactions between users have become the center of attention; the social relationships among consumers have a more significant impact on those consumers’ psychology and behavior than does direct advertising (Lambrecht et al., 2018).

To promote environmentally friendly consumption, many enterprises engage in newsfeed advertising on social media platforms, such as social communication platforms (e.g., WeChat Moments, Weibo), news streaming platforms (e.g., Jinri Toutiao) and short video streaming platforms (e.g., TikTok). The rapid increase in newsfeed advertising on these platforms has been particularly prominent. In addition, newsfeed advertising on the aforementioned platforms conforms to consumer preferences for advertisements given the trend toward more community access, more decision scenarios, and increased behavior fragmentation in the mobile internet era (Martins et al., 2018). The interactive and sharing features of social media platforms increase the willingness of intuitive consumers to build emotional links with others. Furthermore, the sociality of newsfeed advertising on social media platforms can encourage consumers to interact with and propagate information. Proposed by social presence theory, social presence refers to one’s awareness of the existence of the peer and “real ego” when sharing information through social media (Slater and Wilbur, 1997). Social presence can generate a surrounding with warmth and ample interpersonal relationships. The sense of warmth can be perceived by perception of sociality. The sociality of newsfeed advertising facilitates human-computer interaction; meanwhile, users can feel a sense of co-existence when involving in interaction, and generate a sense of warmth (Short et al., 1976; Hassanein et al., 2007). Different from traditional advertising, green advertising can often generate a warm atmosphere. Therefore, compared with traditional newsfeed advertising, green advertising is more able to exhibit the sociality of newsfeed advertising (Luo et al., 2020). At the same time, consumers’ behaviors, such as “like,” “comment” and “forward,” can enhance the sociality of the contents while obscuring transaction attributes, assisting consumers with social relationship construction (He et al., 2018). Compared with traditional advertising, green advertising is more able to fade transaction attributes while presenting the sociality of newsfeed advertising. Previous studies on online green advertising have mainly focused on several-on-one interactions between users and the green advertising, not on online interactions among users who have all viewed the same green advertisement (Liu, 2014; Todri-Adamopoulos and Ghose, 2016). Previous literature on green advertising has investigated the determinants of consumer reactions to green advertisements, such as advertising appeal (Chang et al., 2015; Yang et al., 2015) and target Audience (Leonidou and Hultman, 2011), or has pointed out that the views or expectations expressed by others can affect user reactions to green advertising. However, all these papers simply regard users as the objects of social behaviors; they do not regard users as the initiators of social interactions. Current information streaming media platforms and newsfeed advertisements can be further shared by making it possible for consumers to interact with others (Li and Wang, 2014; Lambrecht et al., 2018). To date, previous research on sociality has focused on information systems (Karel Kreijns, 2007), online communities (Preece, 2001) and virtual worlds (Animesh et al., 2011; Junglas et al., 2013). However, researchers have largely ignored the problems related to the sociality of advertisements, the impacts of green newsfeed advertising on consumers’ green consumption behavior should be examined by scholars. Petty et al. (1983) put forward two routes for changing attitudes, which is referred to as elaboration likelihood model. The two routes can explain the difference of consumers’ decision making by identifying consumers’ involvement. Specifically, compared with consumers with peripheral routes, consumers with central routes pay more attention to information, and conduct comprehensive analysis, consideration, evaluation and induction, which further alters their decision-making behaviors. Thus, consumers with different involvement have different evaluations of green advertising; this is why consumers with different involvement have different reactions and have diverse purchase decision making. Consumers with high involvement have higher degrees of cognition to green information or green products, and thus they identify and appreciate them. On the contrary, since consumers with low involvement have lower degrees of cognition to green information or green products, they often hold dismissive attitudes (Rahman, 2017).

Based on the literature above, we intend to explore the sociality aspect of green newsfeed advertising and its effects on consumers. More specifically, this study aims to answer the research questions as follows: (1) Does the sociality of green newsfeed ads influence consumers’ adoption of new green products? (2) What is the influence mechanism of sociality of green newsfeed ads on consumers’ adoption of new green products? (3) What are the boundary conditions of the above influence mechanism? The goal of this study is to examine the relationships among sociality, receptivity to green advertising, and adoption of green products, based on the Theory of social presence (Short et al., 1976) and elaboration likelihood model (Petty et al., 1983).

In our paper, we propose the sociality of green newsfeed advertising as a mechanism that influences consumer adoption of new green products, and green involvement as a factor that mediates it. Specifically, consumers extensively adopt green products when confronting green newsfeed advertisements with a high degree of sociality; receptivity to green advertising drives the psychological adoption of new green products. In addition, consumer satisfaction with social impulses on social media platforms makes them more accepting of green advertising. When facing green newsfeed advertising with a high degree of sociality, consumers are more receptive to green advertising, which further positively affects their adoption of new green products. However, individuals with different levels of green involvement have different levels of knowledge related to greenness. Thus, consumers participate to different degrees in terms of the sociality of green newsfeed advertising, which impacts their receptivity to green advertising and their real green consumption behavior. We conducted a survey and a quasi-experiment to verify our theory and hypothesis.

The contributions of this paper are threefold. First, the different characteristics of newsfeed advertising have significant impacts on advertisement sharing and willingness to purchase (Lee and Labroo, 2004; Polatidis and Georgiadis, 2015). Based on the theory of social presence, the warmth created by social presence is reflected as one of the characteristics of newsfeed ads: sociality. We extend the related literature to green newsfeed advertising and explore the influence mechanism of sociality of green newsfeed advertising on consumer adoption of new green products. Second, receptivity to green advertising is a precondition for green advertising to impact consumer attitudes and willingness to purchase. We investigate the factors that predispose consumers to be receptive to green advertising in terms of sociality. Third, based on Elm theory, green advertising involvement is proposed in the context of green consumption, we verify the mediating effect of green involvement and untangle the boundary conditions for the adoption of new green products. Advertisers and social media platforms can provide technical support to target users and promote interactions among users. Our paper also helps managers gain insights regarding how to realize the value of new green products by promoting the propagation and popularization of green consumption behaviors.

## Literature Review

### Green Newsfeed Advertising

Green advertising refers to advertisements that typically emphasize the overall environmental benefits associated with the purchase of a specific product, such as reducing greenhouse gas emissions, reducing water pollution, and promoting biological integration (Nyilasy et al., 2014). The theme of green newsfeed advertising is a focus on environmental protection and human health, delivering environmentally friendly messages to and instilling green values in consumers. As the internet has rapidly advanced, many enterprises have chosen to place green advertisements on social media websites, including Facebook, Weibo, WeChat, Wiki, and multimedia sharing websites (e.g., YouTube, TikTok, and Kuaishou). Newsfeed advertising is one of the most effective forms of advertising in the social media environment. Compared with other forms of advertising, newsfeed advertising is considered to be highly effective with a low level of invasiveness (Fulgoni and Lipsman, 2014). An important promotional activity for enterprises is to advertise green consumption behaviors through newsfeed advertising on social media platforms. Well-designed newsfeed advertisements can boost the reliability of green products, promote those green products, extend the market for them and improve brand image.

Iyer and Banerjee (1993) is the earliest work that analyzes green printing advertising. In this study, they first introduce a framework to analyze green advertising, and then analyze samples of green advertising based on this framework. Zinkhan and Carlson (1995) discuss green consumers hold negative attitudes to business, and they are highly likely to possess a negative impression to advertising industry. Some researchers point out that the claims in advertising can affect its credibility. Compared with abstract claims in advertising, specific claims can improve the credibility and memory of information into a higher level (Alniacik and Yilmaz, 2012). This is mainly because consumers do not doubt advertising claims in most cases, and they can easily verify objective and specific information rather than abstract claims. Davis (1993) study the contents of a claim based on an abstract advertising of a shampoo. This study shows that compared with non-specific advertising claims, the specific advertising claims that state environmental benefits can generate significant favorable attitudes and higher willingness to purchase. Yang et al. (2015) find that, when green advertising emphasizes more on altruism, compared with specific advertising claims, abstract advertising claims can intensively improve green consumers’ willingness to purchase. Kim et al. (2021) explore optimized social advertising strategies that can generate consumer engagement with green messages on social media. They investigate the effect of claims’ specification and interest appeal on consumers’ degree of involvement on social platforms and show that consumers’ degree of self-structural moderates the interaction effect of claim specificity and benefit appeals type on consumers’ degree of involvement.

Existing research on green advertising has mainly focused on the effect of environmental involvement, self-construction and directional control on green newsfeed advertising, respectively (Chang et al., 2015; Yang et al., 2015; Kim et al., 2021). However, it is still necessary to investigate the effect of green newsfeed advertising on consumers’ psychology and behavior through empirical methods. Research has proven that, the major characteristics of newsfeed advertising, such as original nature, sociability and dynamics, are significant influencing factors of advertising results. Thus, we will further investigate the boundary conditions and internal mechanisms of how green newsfeed advertising influences consumers’ willingness to purchase green products from the perspective of the characteristics of green advertising.

### Social Presence Theory and Perceived Sociality of Advertising

Social presence, according to Short et al. (1976), refers to the prominence of the opposite when contacting and the prominence of interpersonal relationships brought by the former. This concept is mainly about the relationships between partners and mutual perception when communicating through computers (Gunawardena and Zittle, 1997; Tu and Mcisaac, 2002). Based on Biocca et al. (2003), Shen and Khalifa (2008) define social presence as the awareness of other living people, accompanying the participation of emotion and perception in the social space mediated by computers. According to social presence theory, social presence is the constant awareness of the coexistence of others and the perception of participating in their interactions (Junglas et al., 2013). Traditional advertising, such as banner advertising, does not allow receivers to engage in verbal or nonverbal communication with each other, and thus individuals cannot generate a sense of coexistence. Social presence theory has shown that the interactions between users and information systems can generate a sense of warmth, which is closely correlated with the sense of coexistence, and social perceptions can reflect this feeling. Social presence theory has shown that a sense of warmness can be generated during the interaction between users and information systems. Sociality refers to the degree of generation of social spaces promoted by computer-mediated communication environment though the allowance of social support (Junglas et al., 2013). Traditional online display advertising can be frequently seen on information portals which issue different information, conducting transmission in one-way and one-to-many methods. The designers of traditional online display advertising consider that the audiences are relatively independent, and mainly take the direct effect of audiences’ awareness and memory into consideration (Goldfarb and Tucker, 2011). However, consumers no longer receive messages passively in the social media era; instead, they voluntarily participate in the interaction and transmission of messages (Akpinar and Berger, 2016). Many research has shown that the effect of consumers’ social relationships on consumers’ psychology and behavior is more significant than the direct persuasion of advertising. The more frequently consumers interact with others with regard to advertising, the more easily they generate self-identity and resonance to the brand promoted by the advertising. This facilitates brand image building and brand value transmission. Compared with clicks on advertising, consumers’ social behaviors such as like, comment and forward have more long-term significance (Ljungberg et al., 2017). Socialized transmission can enhance the social characteristic of the contents while obscuring the transaction characteristic, helping the social-relationship construction of consumers. This benefits the brand value construction and brand reputation transmission (He et al., 2018). Embedded in social communication media platforms, newsfeed advertising is endowed with sociality features. Through like, comment and forward, more consumers participate in the transmission of advertising (Lambrecht et al., 2018). For instance, on social communication media platforms such as Facebook, Weibo, and WeChat, the involvement of newsfeed advertising can be improved by intuitive and humor advertising.

Based on previous studies and motivated by the application of the interaction features of newsfeed advertising in reality, we define the sociality of newsfeed advertising as the interaction with other users through newsfeed advertising, thereby satisfying users’ social motivations.

At present, many researchers are still exploring the effect of the sociality of green newsfeed advertising on consumers’ green purchase behaviors. According to the extant research, the sociality of green newsfeed advertising can influence consumers’ sharing behavior, attitudes and comments as well as willingness to purchase. Thus, this study will investigate the effect of the sociality of green newsfeed advertising on consumers’ attitude and willingness to purchase.

### Receptivity of Green Advertising

Green advertising receptivity refers to the degree to which green newsfeed advertising is received on social media platforms (Bailey et al., 2016). The receptivity of advertising measures the effectiveness of the advertisement, including the exposure rate and content design. Previous research has revealed that green advertising affects consumer consumption tendencies, generating willingness to purchase among consumers. Green advertising promotes information about green energy-saving products to consumers through television channels, Weibo and Tik Tok (Zhang et al., 2019). When exposed to green advertising on social media platforms, consumers’ perceptions of and judgments about green advertising affect their attitudes toward green advertising and their beliefs about green brands or green products (Bailey et al., 2016). Existing studies have shown that receptivity to green advertising is a precondition for environmentally friendly advertising, information or sales promotion activities to influence consumer attitudes and willingness to pay. Sun et al. (2020) integrate models of the impacts of receptivity to green newsfeed advertisements on consumer willingness to purchase. They also examine the moderating effect of regulatory focus.

Although green advertising on social media platforms promotes environmental purchases, consumers do not make such purchases immediately after viewing green advertisements. Moreover, receptivity to green advertising varies from person to person. Previous literature has shown that there are individual differences in the effect of advertising appeals across consumers (Schuhwerk and Hagius, 2013). Tucker et al. (2012) illustrate how individual characteristics possibly affect consumers’ receptivity to the claims promoted by environment-themed advertising. The experiment compares the promotion strategy between strong green product claims and weak green product claims. The results show that consumers who have a positive attitude to environmental protection can also accept all conditions.

According to existing research, consumers’ receptivity of green advertising can essentially affect their psychological activities and decision-making behaviors. Thus, this paper will investigate the effects of the sociality of green advertising on consumers’ willingness to pay in the social-media background; meanwhile, we conduct the analysis by regarding the receptivity of green advertising as a mediating mechanism.

### Adoption of New Green Products

The adoption of new green products refers to consumers’ willingness and decision to purchase environmentally friendly products. Previous literature has studied how promotion activities on social media platforms influence consumers’ green purchase behaviors. Some researchers point out that social media marketing has a significant positive impact on consumers’ willingness to purchase (Froehlich, 2009; Hynes et al., 2016). However, few researchers show that social media marketing influences consumers’ willingness to purchase green products only by some mediating factors (Dopaço et al., 2018).

Social media allows users to communicate with people who possess similar interests. Social media marketing benefits advertisers since consumers choose to communicate with people who have similar lifestyles on social media platforms (Lee et al. 2018). Marketing personnel can search for sustainable consumer groups through social media platforms and recommend green products to them. Furthermore, social media facilitates the generation and transmission of information (Lee et al. 2018). Users can release and share their original contents and viewpoints on social media platforms. Thus, it can be an opportunity for enterprises to generate online marketing strategies through social media marketing. This is because consumers acquire information through social media platforms, and make purchase decisions based on the acquired information. Moreover, because of users’ personal characteristics (e.g., interaction, social networks, and personal relationships), social media marketing is a reliable advertising tool. Researchers have shown that social media marketing has a great impact on consumers’ willingness to purchase (Froehlich, 2009; Hynes et al., 2016).

Existing studies have emphasized the importance of social media platforms to consumers’ adoption of green products. Our paper contributes to literature by analyzing how green newsfeed advertising improves consumers’ receptivity of green advertising and further influences their adoption of green products.

### Elaboration Likelihood Model and Green Involvement

In elaboration likelihood model, Petty et al. (1983) consider the difference between the degree of individual’s information refinement and cognitive input; moreover, there are two routes of information processing, that are, central route and peripheral route. ELM theory points out that the change of individual information processing way or attitude can be divided into central route and peripheral route for explaining consumers’ information processing behavior. Based on information receivers’ input degree is different: the central route has a more intensive control desire of information, and often spends more energy and time deeply analyzing and comprehending information; peripheral route does not focus much on information, and only needs to spend less energy and time understanding peripheral hints. Rokon U Zzaman et al. (2020) find that consumers will be affected by involvement when searching for shopping information and judging and analyzing relevant information. Based on ELM theory, under green consumption scenarios, green involvement reflects consumers’ concerns about the input of green advertising or green products as well as concerns about information on environmental protection. Obviously, consumers with high green involvement are driven by central routes, while consumers with low green involvement are powered by peripheral routes.

Different individuals have different concerns about specific green advertisements or green products; i.e., consumers participate in specific activities to different degrees. These differences affect the psychological status and behaviors of an individual. Sheng et al. (2020) study the effects of genres of green products, appeals of green advertising and impression management incentives on intention to purchase green products based on ELM theory. Souza and Taghian (2005) show that consumers who have high levels of green involvement are willing to believe information about green products, which can further generate positive attitudes toward green products. Some studies have shown that information about environmental protection has a more positive impact on consumers who have high levels of green involvement, while consumers who have low levels of green involvement do not pay much attention to such information (Martin and Timothy., 2013). However, some research has drawn inconsistent conclusions. Schuhwerk and Hagius (2013) find that there are no differences in the purchase decisions of consumers with high levels of green involvement. However, for consumers with low levels of green involvement, there are significant differences. Compared with information about nongreen products, information about green products has a more positive impact on consumers’ purchase decisions. Vlieger et al. (2013) find that consumer beliefs about the credibility of information on green products has a negative impact on their green involvement; i.e., higher levels of green involvement lead to lower perceptions of credibility.

Batra and Ray (1986) show that when faced with green advertising, consumers’ green involvement affects the effectiveness of green appeals. Compared with consumers with low levels of green involvement, consumers with high levels of green involvement have more positive attitudes toward green advertisements. Matthes et al. (2014) find that green involvement impacts the publicity of green advertising and that green involvement has a significant impact on consumer attitudes toward brands and on their purchase decisions. Xu et al. (2015) find that the decision-making process for consumers with high levels of green involvement is much more complicated than that for consumers with low levels of green involvement. These two segments have different reactions to information and have different levels of receptivity. Souza and Taghian (2005) find that green involvement can affect the impact of advertising appeals on attitudes toward advertisements and perceived value. Consumers with low levels of green involvement are more inclined to ignore green appeals.

This study investigates whether individuals’ different green involvement has different impacts on the relationship between the sociality of green newsfeed advertising, receptivity of green advertising, and adoption of green new product based on ELM theory. Therefore, we mainly focus on discussing how green newsfeed advertising influences consumers’ receptivity of green advertising and willingness to purchase given different green involvement.

### Summary

First, the existing literature on newsfeed advertising has mainly focused on the effects of characteristics (Fan et al., 2018), mechanisms (Wojdynski and Evans, 2016), and outcomes (Harms et al., 2017). However, very few researchers have focused on the psychological and behavioral mechanisms underlying newsfeed advertising. Our paper focuses on newsfeed advertising and investigates the mechanisms by which it affects consumer behavior based on the theory of social presence.

Second, regarding the characteristics of newsfeed advertising on social media platforms, the sociality of newsfeed advertising can decrease the negative perceptions of receivers to some extent and can affect consumer avoidance of advertising (Fan et al., 2018). Our paper further examines the effects of the sociality of newsfeed advertising on consumers.

Third, existing research has investigated the mechanisms by which green newsfeed advertising influences consumers’ environmental purchase behaviors (Batra and Ray, 1986; Sun et al., 2020). However, few researchers have regarded sales promotion activities on social media platforms as signals and stimuli that affect consumer cognition or investigated the mechanisms influencing environmental purchase behaviors. Based on this, our paper investigates the impact of green newsfeed advertising on consumer adoption of new green products in terms of the social media context. We adopt the concept of receptivity to green advertising and study the impacts of receptivity to green advertising on the adoption of new green products. Additionally, we consider the negative interaction effects of green involvement as well as receptivity to green advertising on the adoption of new products and investigate the psychological mechanisms underlying and boundaries on the adoption of new green products. This is a strong contribution to the existing literature.

## Conceptual Framework and Research Hypothesis

### The Sociality of Green Newsfeed Advertising and the Adoption of New Green Products

According to social presence theory, social presence refers to the extent to which an entity is perceived as a “real person” and the perceived level of connection with others during the process of using media to communicate (Hassanein et al., 2007). An environment with high social presence makes people more inclined to connect with others rather than ignore them. Hassanein and Head (2004) showed that the inclusion of elements required for customer social interactions in a website can enhance customers’ sense of social presence, thus improving perceived usefulness, trust and pleasure, and thus consumers’ desire to buy. The perceived sociality of infomercial green ads can reflect this sense of social presence.

In the context of social network, the sociality of green newsfeed advertising is mainly reflected in that users themselves are not only the receiver of green advertising, but also the disseminator of green advertising. Through this communication process, they form social contacts with other users, enhance social communication and enhance consumers’ green products adoption. Buttons such as “like,” “comment” and “forward” are displayed below the green newsfeed advertising. Thus, users can publish and share their original content and views on social media, or infer the views of other audiences on the green ads through these indicators, so as to improve users’ perception of the sociality of such ads, so as to enhance consumers’ purchase desire.

Social media marketing is an opportunity for enterprises to formulate online marketing strategies, because consumers can obtain information through social media and then make purchase decisions based on the information. Therefore, in the face of the sociality of green newsfeed ads, the individual’s cognition of green products or green behavior may be clearer in the social situation, and produce a sense of belonging in the process of communicating with other users, which will induce the diffusion behavior and green purchase behavior of green advertising.

Combining the above analysis, we propose the following hypothesis:

*H1*: The sociality of green newsfeed advertising has a positive impact on the adoption of new green products.

### The Mediating Effect of Receptivity to Green Advertising

Green newsfeed ads contain not only information related to green products or brands but also messages left by the green ad audience, such as user comments. The number of likes, shares and audience responses can be used as indicators to evaluate the perceived value of the green ads to others and their influence over other audience members’ perceptions of these green ads. Sociality allows for direct verbal and nonverbal communication between audiences. According to social presence theory, sociality can create a sense of warmth among users (Cheikhammar and Barki, 2014) while also enhancing their perceived pleasure and creating a sense of close belonging among individuals (Karel Kreijns, 2007). Green newsfeed ads support social interactions among users through functions such as liking, sharing and replying, thus satisfying users’ social impulses to a certain extent and improving their experience; therefore, the sociality of green social media newsfeed ads can reduce the audience’s negative perceptions of green ads and improve their green ad receptivity to a certain extent.

When consumers are exposed to green ads on social media, they experience certain feelings and form certain judgments, which influence their attitudes toward the green ads themselves and their beliefs about green brands or green products (Bailey et al., 2016). Green advertising can enhance consumers’ understanding of green products, promote green products, and increase their willingness to purchase green products. When consumers choose a product by excluding alternatives, they place more emphasis on green attributes (Irwin and Naylor, 2009). When consumers are aware of the green attributes of products due to green advertisements, they are more likely to purchase those products (Hartmann and Apaolaza-Ibanez, 2013). Previous studies have shown that green advertising aims to influence consumers’ purchasing behavior by encouraging them to purchase products with green labels and making them aware of the positive effects of their purchases on themselves and the environment (Rahbar and Wahid, 2011). Therefore, the higher the consumer’s receptivity to green advertising, the higher their understanding of green products may be and the higher their willingness to purchase green products (Chang et al., 2015).

From the above analysis, we propose the following hypothesis:

*H2*: Green advertising receptivity mediates the relationship between the sociality of green newsfeed ads and the adoption of new green products.

### Green Involvement as a Moderator

The elaboration likelihood model proposed by Petty et al. (1983) pointed out that when target users process the information content of advertisements, their investment level will affect their attitudes towards advertisements and brands. Using ELM model to process information is related to the time and experience invested. When there is time and energy to process information finely, consumers will process information through the central route; Individuals who lack time or energy tend to process information through peripheral route. The level of involvement reflects the degree of individual participation in processing information. The higher the level of involvement, the higher the degree of participation in processing information, and will tend to process information more elaborate.

Green involvement can influence the attention consumers give to green products, and it is an important factor to consider when studying the intention to purchase green products. Consumers with high levels of green involvement are willing to collect more descriptive information on product characteristics and conduct detailed and specific analyses. The communication among users in green newsfeed advertising can help consumers understand the information related to green characteristics and make more comprehensive judgments. Therefore, green involvement can improve consumer receptivity to green advertising and promote consumers’ intentions to make green purchases. In contrast, for consumers who do not know much about green products, the interactions among users of green advertising are not attractive and may even cause consumers to perceive target obstacles and advertising clustering, resulting in advertisement avoidance. Therefore, due to the different levels of green involvement among consumers, this interaction can have an impact on consumers’ receptivity to green advertising and their green purchase intentions (Chebat and Picard, 1985). Therefore, for consumers with a high level of green involvement, the positive effect of the sociality of green newsfeed advertising on receptivity to green advertising is enhanced; for consumers with a low level of green involvement, the positive effect of the sociality of green newsfeed advertising is weakened.

Existing research has shown that green information has a more positive impact on consumers with high levels of green involvement, while consumers with low levels of green involvement pay less attention to green product information (Chebat and Picard, 1985). Consumers with high levels of involvement are more willing to actively participate in green newsfeed ad interactions to evaluate the product and thus make decisions. Consumers’ own understanding of products affects their decision-making, and consumers who are more informed about green products are less likely to believe a firm’s one-sided green advertising. Low-involvement consumers invest less attention to the information related to green advertising and know less about green products. Consumer evaluations of green advertising and the receipt of more product information through green newsfeed advertising hinder the fluency of information processing (Lee and Labroo, 2004) and affect users’ green purchase intentions. Therefore, the sociality of green newsfeed ads is more effective among consumers with high levels of green involvement, while the positive effect of the sociality of green newsfeed ads is diminished at low levels of green involvement.

Combining the above analysis, we propose the following hypotheses:

*H3*: The degree of a consumer’s green involvement moderates the relationship between the sociability of green newsfeed advertising and the adoption of new green products.

*H3a*: The positive impact of the sociability of green newsfeed advertising on the adoption of new green products is stronger among consumers with high levels of green involvement.

*H3b*: The positive impact of the sociability of green newsfeed advertising on the adoption of new green products is weaker among consumers with low levels of green involvement.

*H4*: The degree of a consumer’s green involvement moderates the relationship between the sociability of green newsfeed advertising and consumer receptivity to green advertising.

*H4a*: The positive impact of the sociability of green newsfeed advertising on consumer receptivity to green advertising is stronger among consumers with high levels of green involvement.

*H4b*: The positive impact of the sociability of green newsfeed advertising on consumer receptivity to green advertising is weaker among consumers with low levels of green involvement.

### Conceptual Model

**Figure 1:**
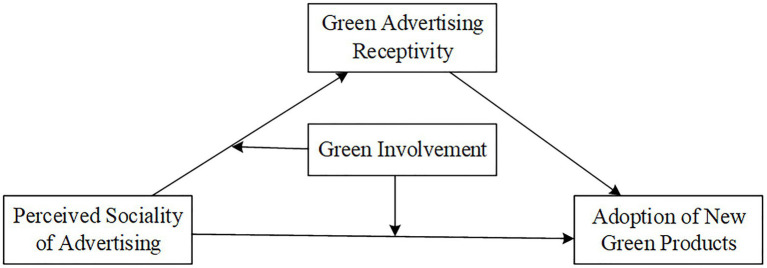
Conceptual model.

A majority of information streaming media platforms such as WeChat, Tik Tok stimulate consumers to actively participate in advertising interaction and actively carry out the secondary dissemination of advertising through the sociality of newsfeed advertising. Our study explores factors that influence Adoption of New Green Products. The research model proposed in this study is shown in Figure 1. Based on the theory of social presence and the related research of green advertising, social presence is defined by social psychology. Social presence pays attention to the social attributes such as interaction or communication, and regards them as a sense of warmth. The sociality of newsfeed advertising can reflect the aforementioned sense of warmth, satisfy consumers’ needs for communication by interactive features of newsfeed advertising, and improve consumers’ willingness to use green products by perfecting their advertising experience. Sociality will positively affect the receptivity of green newsfeed ads, and then affect the adoption of new green products by consumers. In the ELM theory, motivation and ability are the key factors that influence the elaboration likelihood by information receivers, with the central route usually being used when motivation is stronger and the peripheral route conversely. Motivation consists of three factors: involvement, variety of arguments, and individual cognitive needs. Studies have shown that the higher the level of involvement, the stronger the motivation to process the information (Krugman, 1965), consumers are more willing to engage with the social features of green newsfeed ads, which in turn influences their attitudes and behaviors toward green ads and green products. Therefore, in the case of high involvement, the interaction of involvement and sociality will have an impact on green product adoption and green ad receptivity. So green advertising involvement will play a moderate role.

Based on the discussion above, the conceptual model of this study can be built as shown in Figure 1.

## Study 1

### Participants, Procedure and Measures

One hundred forty students from a school in Guangxi were invited to participate in this study. Of those students, 68 were male, and 72 were female (M_age_=25.53, SD=5.139).

Study 1 had a one-way (sociality of green newsfeed advertising: high vs. low) between-subject design. The participants were randomly divided into two groups (high vs. low). There were 70 participants in each of the two groups. To better motivate the participants to complete the experiment, the participants who participated seriously in the experiment received a reward of 3 RMB each.

China has the most social media users in the world. In China, social media are classified into microblogging sites (e.g., microblogs and blogs), social networking sites (e.g., QQ and WeChat), video and photo sharing sites (e.g., TikTok and Kwai), and community exchange sites (e.g., Post Bar and Zhihu). Since the experimental participants are school students who are familiar with microblogs, which support interactions among users and their nonsocial media friends, Sina Weibo was chosen as the social platform on which the infomercial was distributed in this experiment.

Participants in the highly social green newsfeed ad group read a green ad on Weibo with the ability to like, retweet and comment. The green newsfeed ad was in the same format as the native Weibo content, with only the word “ad” in the upper right corner, and advertised a product made from recycled materials. The number of likes, retweets, and consumer comments on the green ad, as well as the interactions among consumers, were displayed below the ad. Participants in the nonsocial green newsfeed ad group read a green ad without the ability to like, retweet, or comment; in all other ways, the form and content of the green ad were the same as those of the highly social green newsfeed ad. Participants in both groups were presented with textual materials explaining the concepts of green infomercial advertising and green advertising.

After reading the materials, the participants completed a questionnaire on the sociality of green newsfeed ads, receptivity to green ad and the adoption of new green products. Finally, participants were asked to fill in their demographic information such as gender and age. All items were assessed using a 7-point Likert scale (1=strongly disagree; 7=strongly agree). We used SPSS 26.0 to conduct exploratory factor analysis on the data of each variable. The results showed that the factor load of all variable measurement indicators was greater than 0.700, the eigenvalue was greater than 1, the Cronbach’s α value for the green newsfeed ad sociality scale (Han and Drumwright, 2017) was 0.867, the Cronbach’s α value for the new green product adoption scale (Sun et al., 2018) was 0.931, and the Cronbach’s α for the green ad receptivity scale (Paço and Reis, 2012) was 0.878. the Cronbach’s a value of each variable was more than 0.867, and the KMO coefficient was more than 0.775, indicating that the measured variables had high reliability and validity and were suitable for formal experiments.

### Analysis and Results

#### Manipulation Test

To test the success of the manipulation of the sociality of the green newsfeed ads, we conducted an independent sample t-test on the perceived sociality scores of the participants in the presence of social and nonsocial green newsfeed ad group. The results show that there was a significant difference in the level of perceived sociality scores between the low-sociality ad (M_low-sociality_=4.42, SD=0.93) and the high-sociality ad (M_high-sociality_=5.28, SD=0.89), t(60)=−5.58, *p*<0.001. These results were consistent with expectations, suggesting that the manipulation of the advertising claims was successful.

#### Hypothesis Testing

The results of the one-way ANOVA indicated that the high-sociality group was significantly more receptive to the green ad than the low-sociality group [M_high-sociality_=5.62, M_low-sociality_=4.72, *F*(1, 138)=30.38, *p*<0.001]. In addition, the high-sociality group participants had significantly lower scores on the adoption of new green products scale than the low-sociality group [M_high-sociality_=5.74, M_low-sociality_=4.86, F(1, 138)=34.66, *p*<0.001].

Next, referring to mediation analysis model (Model 4), using the bootstrapping method to test the mediation model (Preacher et al., 2007; Hayes, 2013). As shown in Table 1, the total effect of the sociality of green newsfeed ads on consumer adoption of new green products was significant (0.58, 1.16), and the effect size was 0.87. The mediating effect of receptivity to green ads was significant (LLCI=0.40, ULCI=0.89, excluding 0), and the mediating effect was 0.63. In addition, the sociality of the green newsfeed ads (high vs. low) had a significant effect on the adoption of new green products after controlling for receptivity to green ads (LLCI=0.02, ULCI=0.03, excluding 0), and the effect size was 0.24. The direct effect (0.24) and mediating effect (0.63) accounted for 27.61 and 72.39% of the total effect (0.87), respectively. Green ad receptivity partially mediated the relationship between the sociality of green newsfeed ads and the adoption of new green products.

**Table 1:** Total effects, direct effects and mediating effects.

	Effect	Boot SE	Boot LLCI	Boot ULCI	Relative effect
Total effect	0.87	0.15	0.58	1.16	
Direct effect	0.24	0.10	0.02	0.03	27.61%
Mediating effect	0.63	0.13	0.40	0.89	72.39%

### Discussion

In Study 1, two green newsfeed ads with different levels of sociality promoting recycled items were presented to the participants, preliminarily demonstrating that the effects on consumer adoption of new green products varied with the sociality of the green newsfeed ads. The results show that the two groups had significantly different intentions to adopt new green products and that consumers had a higher willingness to adopt new green products when facing green newsfeed advertisements with high levels of sociality. The direct effect of the sociality of the green newsfeed ads on consumer adoption of new green products was significant, consistent with H1. In addition, we explored the mechanism by which the sociality of green newsfeed ads influenced the adoption of new green products. The differences in perceived sociality (high vs. low) affected the participants’ receptivity to the green ad, which affected their adoption of the new green product. The results showed that, compared with ads with low levels of sociality, highly social green newsfeed ads lead consumers to become more receptive to green ads and thus result in increased consumer intentions to adopt green products, which confirmed that green ad receptivity plays a partially mediating role in the relationship between the sociality of green newsfeed ads and consumer adoption of new green products. Hypothesis H2 is verified. Next, using a questionnaire, Study 2 used a phosphate-free detergent as the subject of green advertising to verify the direct and partial mediating effects and to further verify whether the above influencing mechanism is moderated by green involvement.

## Study 2

### Sample and Data Collection

This study used a questionnaire to collect data from Chinese consumers to test the research hypotheses. The online questionnaire was posted on Wenjuanxin,^1^ a platform that provides functions equivalent to Amazon Mechanical Turk, from July 15 to August 15, 2021 and was forwarded inside social media platforms such as Weibo, WeChat and QQ. The questionnaire shows a green advertisement based on the newsfeed ads on Sina Weibo; the ad focused on a phosphate-free detergent. The newsfeed ad included the functions of liking, retweeting and commenting to facilitate consumer interactions, with the number of likes, retweets and consumer reviews of the green ad and product, as well as the interactive content between consumers, on display below the advertisement. This study also contained the same explanatory text about green newsfeed advertising and green advertising as in Study 1. The green involvement scale used was Zaichkowsky (1985) scale, and the other variable scales were the same as in Study 1, with a 7-point Likert scale (1=strongly disagree, 7=strongly agree) for all question items. The Cronbach’s α value for green involvement was 0.852.

One hundred adult social media users volunteered to participate in the online study in exchange for 3 RMB. A total of 100 questionnaires were received from 55 males and 45 females (M_age_=24.98, SD=5.28).

### Measurement Model and Analysis

This study used R 4.0 and SPSS 26.0 to analyze the data. The results of the confirmatory factor analysis (CFA) for the overall fit indices for the measurement model were as follows: *χ^2^/df*=1.69, *p*<0.001, CFI=0.94, TLI=0.92, IFI=0.94, PNFI=0.71, and RMSEA=0.08. These results indicate that the fit of the measurement model met the threshold values. The reliability and validity statistics are shown in Tables 2 and 3.

**Table 2:** Confirmatory factor analysis (CFA) results for the measurement model.

Construct	Item	Loading	Cronbach’s α	Composite reliability	AVE
Sociality of Green Newsfeed Advertising	SGNA1	0.818	0.899	0.901	0.647
	SGNA2	0.821			
	SGNA3	0.759			
	SGNA4	0.840			
	SGNA5	0.767			
Receptivity to Green Advertising	GAR1	0.728	0.821	0.822	0.536
	GAR2	0.723			
	GAR3	0.732			
	GAR4	0.747			
Green Involvement	GI1	0.784	0.852	0.855	0.596
	GI2	0.770			
	GI3	0.739			
	GI4	0.802			
Adoption of New Green Products	GPA1	0.803	0.843	0.842	0.573
	GPA2	0.774			
	GPA3	0.745			
	GPA4	0.719			

**Table 3:** Means, standard deviations (SDs), and correlations.

	Mean	SD	SGNA	GAR	GI	GPA
SGNA	5.475	0.892	0.804			
GAR	4.528	0.745	0.643^**^	0.732		
GI	2.694	0.736	−0.138	−0.174	0.772	
GPA	5.590	0.954	0.488^**^	−0.533^**^	−0.240^*^	0.757

* *p*<0.05;

** *p*<0.01.

In addition, all of the constructs in this study had good discriminant validity, as the correlation coefficient between each pair of constructs was smaller than the square root of the AVE values of the two constructs (Fornell and Larcker, 1981). These results provide strong support for the adequacy of the reliability, convergent validity, and discriminant validity of the constructs.

Since this study used single-source data, it was necessary to check for common method bias (CMB). We used the method proposed by (Harman, 1976), and the results showed that the variance explained by the first principal component was 32% of the total cumulative variance, which is less than 50%, basically indicating that there is no serious common method bias. Additionally, the variance inflation factors of all predictor variables were tested and found to be no higher than 1.446, so there is no serious problem with multicollinearity.

### Moderating Effect Test

**Figure 2:**
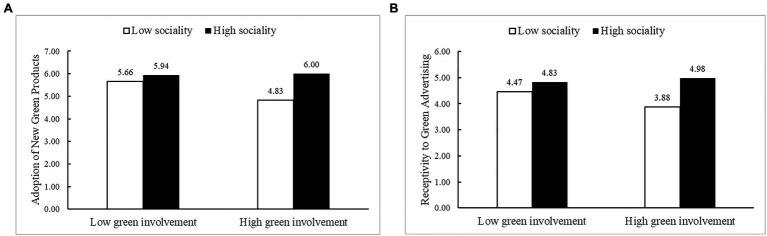
Means of the variables at different levels of green involvement. **(A)** Means of the adoption of new green products at different levels of green involvement. **(B)** Means of the receptivity to green advertising at different levels of green involvement.

An ANOVA was conducted with the adoption of new green products as the dependent variable. The results showed that the sociality of the green newsfeed ad [*F*(1, 96)=18.40, *p*<0.001, η^2^=0.16] and its interaction with green involvement [*F*(1, 96)=6.94, *p*=0.01, η^2^=0.07] were significant, as was the main effect of green involvement [*F*(1, 96)=5.20, *p*=0.025<0.05, η^2^=0.05]. An ANOVA with receptivity to green ads as the dependent variable showed that the main effect of the sociality of green newsfeed ads [*F*(1, 96)=34.51, *p*<0.001, η^2^=0.26] and its interaction with green involvement [*F*(1, 96)=8.81, *p*=0.04, η^2^=0.08] were also significant and that the main effect of green involvement was not significant [*F*(1, 96)=3.05, *p*=0.08>0.05, η^2^=0.03]. The mean values of the variables at different levels of green involvement are shown in Figure 2. Figure 2 shows that the variation in both the mean values of receptivity to green advertising and the adoption of new green products are similar for different levels of green involvement.

### Moderated Mediating Effect

There were no significant effects of gender, age, household income or education on the adoption of new green products. A moderated mediating model was established to test whether the direct and mediating effects of the sociality of green newsfeed ads on consumer adoption of new green products was moderated by the consumer’s level of green involvement. As shown in Table 4, the sociality of green newsfeed ads had a significant effect on receptivity to the green ad (*β*=0.46, *t*=6.61, *p<* 0.001), which had a significant effect on new product adoption (*β*=0.33, *t*=2.75, *p*<0.01). The interaction between green involvement and the sociality of green newsfeed ads had a significant effect on new product adoption (*β*=0.23, *t*=2.68, *p*<0.01), and the interaction between green involvement and the sociality of news feed ads had a significant effect on receptivity to the green ad (*β*=0.26, *t*=3.67, *p*<0.001).

**Table 4:** Moderated mediation model test.

	Receptivity to green advertising			Adoption of new green products		
	*β*	*se*	*t*	*β*	*se*	*t*
Sociability of Green Newsfeed Advertising	0.46	0.07	6.61^***^	0.37	0.09	3.94^***^
Green Involvement	−0.12	0.08	−1.44	−0.30	0.10	−3.13^**^
Receptivity to Green Advertising				0.33	0.12	2.75^**^
Sociability×Green Involvement	0.26	0.07	3.67^***^	0.23	0.09	2.68^**^
R-sq	0.41			0.50		
F	22.36			24.15		

** *p*<0.01;

*** *p*<0.001.

Then, the mediation model (Model 8) was verified by using the same method used in Study 1(Hayes, 2013). As shown in Table 5, for high levels of green involvement, the direct effect of the sociality of green newsfeed ads on the adoption of new green products was significant (LLCI=0.31, ULCI=0.78, excluding 0) with an effect size of 0.54; the mediating effect of receptivity to the green ad on the relationship between the sociality of green newsfeed ads and the adoption of new green products was also significant (LLCI=0.45, ULCI=0.77, excluding 0), and its effect size was 0.61. For the low-sociality advertisement, the direct effect of the sociality of the green newsfeed ad on the adoption of new green products was not significant (LLCI=−0.02, ULCI=0.41, including 0); the mediating effect of receptivity to the green ad was significant (LLCI=0.06, ULCI=0.41, excluding 0), and its effect size was 0.23. The results of the moderated mediation model verify H3 and H4.

**Table 5:** Mediating effect in different green involvement levels.

	Green involvement	Effect size	Boot SE	Boot LLCI	Boot ULCI
Direct effect	Low	0.20	0.11	−0.02	0.41
	High	0.54	0.12	0.31	0.78
Moderated mediating effect	Low	0.23	0.09	0.06	0.41
	High	0.61	0.08	0.45	0.77

### Discussion

Study 2 verified the boundary conditions for the main effect of the sociality of green newsfeed ads on the adoption of new green products and the mediating effect of receptivity to green ads. The results reconfirmed that the sociality of green newsfeed ads significantly affect consumer adoption of new green products. Moreover, the sociality of green newsfeed ads affected consumer receptivity to those green ads, and receptivity to the green ads had a significantly positive effect on consumer adoption of the new green product (Li and Wang, 2014). Study 2 used a questionnaire to investigate how the positive effects of the sociality of newsfeed ads on receptivity to green ads and the adoption of new green products varied under different levels of green involvement. The results of the study show that when consumers report high levels of green involvement, the positive effect of green ad sociality on receptivity to green ads and on the adoption of new green products increased, while for consumers who reported low levels of green involvement, the positive effect of green ad sociality on receptivity to green ads and the adoption of new green products was alleviated. The level of green involvement moderates both the direct effect of green ad sociality on green product adoption and the mediating effect on receptivity to green ads, verifying H3 and H4.

## General Discussion

### Research Conclusion

We investigated how the use of newsfeed technology in green advertising affects consumer adoption of new green products. Based on advertisements for two green products, a product made from recycled materials and a phosphate-free laundry detergent, we used a combination of experimental and survey methods to explore the mechanisms underlying and boundary conditions for the effect of the social attributes of green infomercial advertising on consumer adoption of new green products.

We captured user perceptions of the social attributes of newsfeed ads based on one of the technical characteristics of newsfeed ads: sociality (Lambrecht et al., 2018). The sociality of green newsfeed ads affects consumer adoption of new green products, and receptivity to green ads partially mediates this effect. Study 1 confirmed this direct effect and the partial mediation of receptivity to green ads. Through a questionnaire-based survey, Study 2 confirmed that the direct and mediating effects of receptivity to green ads on the sociality of green newsfeed ads and on the adoption of new green products are moderated by consumers’ green involvement. High levels of green involvement enhance the positive effects of the sociality of the infomercial on receptivity to green ads and new green product adoption, while low levels of green involvement alleviate the positive effects of the sociality of newsfeed ads on receptivity to green ads and new green product adoption.

### Theoretical Contributions

First, this study advances the empirical research on green newsfeed advertising and expands the scope of research on consumer adoption of new green products. Newsfeed ads on social media have their own unique characteristics: they are consistent with the native content in terms of form and function while meeting the need for interaction among users (Lee and Labroo, 2004; Polatidis and Georgiadis, 2015). In social presence theory, consumers obtain a sense of social presence through interactive functions when using social media, and this sense of social presence gives consumers a sense of warmth, which in this study is reflected in sociality. In this paper, the direct effect of the sociality of green newsfeed ads on the adoption of new green products is verified through experimental and survey methods.

Second, existing research has established different models for the mechanism by which receptivity to green ads influences the intention to purchase green products (Li and Wang, 2014; Sun et al., 2020), neglecting the study of the antecedent influences on receptivity to green ads in the context of social media. Therefore, this study extends the study of the influence of green ads on consumer adoption of new green products to newsfeed ads on social media, focusing on the social attributes of newsfeed ads and exploring the influence of the sociality of newsfeed ads on the adoption of new green products, thus theoretically and empirically exploring how the sociality of newsfeed ads affects consumers’ green consumption behavior and verifying the effect of the sociality of newsfeed ads on the adoption of new green products. We also verify the mechanism underlying the effect of sociality on green product adoption.

Third, in the green marketing context, the ELM theory suggests that there are differences in the processing of green advertising messages and the formation of consumer attitudes, the different levels of attention given to green ads by consumers affect their psychology and their green consumption behaviors (Wang et al., 2018). In this study, levels of green involvement were introduced into the research framework to determine the boundary conditions for the effectiveness of the sociality of green newsfeed ads. The study found that green involvement among consumers moderates the effect of the sociality of newsfeed ads on receptivity to green ads and on new green product adoption, and the boundary conditions for the main effect and mediating mechanism were determined in order to build a more in-depth and clearer framework in the theoretical and applied fields.

### Managerial Implications

Social advertising is a very effective form of advertising in which advertisers can provide social functions within their ads and benefit from spontaneous interactions among consumers. When launching newsfeed ads on social media, advertisers can choose social media platforms with full user-interaction features as their advertising platform to avoid the negative impacts of low sociality. Advertisers and social media providers should deepen their understanding of the traditional mechanisms underlying internet advertising and, on this basis, make better use of sociality in their green newsfeed ads to improve the user experience and enhance their competitiveness.

Enterprises should pay attention to the publicity of their green advertising. Publicity should be based on the consistency of the relationships among the attributes, values and characteristics of their green product, which will help to shape and strengthen consumers’ feelings or ideas about that green product. In green advertisements, specific information should be transmitted instead of general information, such as information about the specific environmental benefits produced by the green products being advertised, as this is conducive to improving consumer receptivity to the green advertisements and enhancing consumer understanding of the green products.

Enterprises should also focus on targeting their marketing on social media to different consumer groups. On social media, more green product advertisements should be recommended to consumers with high levels of green involvement to stimulate their intention to make green purchases. For consumers with low levels of green involvement, enterprises should recommend green advertisements to specific consumers on social media in order to develop and educate consumers about green labels and green products so that consumers can clearly see the unique advantages of green products, develop the knowledge and skill to purchase green products, and increase their green involvement.

### Limitations, and Future Research

The limitations of our study include the following four issues. (1) The theoretical model does not account for the characteristics of the social media. Consumer behavior on social media is closely related to the consumers’ motivations, and future research can combine social media features with user models to examine audience perceptions of green newsfeed ads in different media environments and deepen the understanding of green infomercial applications. (2) To increase the practicality of this study’s managerial insights, future research can add other characteristics of green newsfeed ads to the research model, such as the relative positions of the green ads. The location of embedded ads affects the attention users give to the ads (Liao et al., 2017), so future research can control for the relative position of green ads in the newsfeed, such as the front or middle part of the news feed, as a way to derive more specific guidance on green newsfeed advertising practices. (3) This study did not examine the strength of the relationships among users when examining the sociality effect, and future research could further examine the effect of the strength of user relationships on the relationship between sociality and audience perceptions of green advertising. (4) This study lacks a specific limitation for green products. Future research may investigate specific green products and compare consumer purchase intentions across different green products.

## Data Availability Statement

The raw data supporting the conclusions of this article will be made available by the authors, without undue reservation.

## Ethics Statement

Ethical review and approval was not required for the study on human participants in accordance with the local legislation and institutional requirements. Written informed consent for participation was not required for this study in accordance with the national legislation and the institutional requirements.

## Author Contributions

XC, XW, and XH contributed to conception and design of the study and wrote sections of the manuscript. XC organized the database and wrote the first draft of the manuscript. XW performed the statistical analysis. All authors contributed to the article and approved the submitted version.

## Funding

This work was supported by the National Natural Science Foundation of China (71872055 and 72062001).

## Conflict of Interest

The authors declare that the research was conducted in the absence of any commercial or financial relationships that could be construed as a potential conflict of interest.

## Publisher’s Note

All claims expressed in this article are solely those of the authors and do not necessarily represent those of their affiliated organizations, or those of the publisher, the editors and the reviewers. Any product that may be evaluated in this article, or claim that may be made by its manufacturer, is not guaranteed or endorsed by the publisher.

## Footnotes

^1^ https://www.sojump.com/
